# A Novel Program Scheme for Z-Interference Improvement in 3D NAND Flash Memory

**DOI:** 10.3390/mi14040896

**Published:** 2023-04-21

**Authors:** Jianquan Jia, Lei Jin, Xinlei Jia, Kaikai You

**Affiliations:** 1 Institute of Microelectronics, Chinese Academy of Sciences, Beijing 100029, China; 2 University of Chinese Academy of Sciences, Beijing 100049, China

**Keywords:** 3D NAND, adjacent gate pass voltage, charge-trapping memory, cell-to-cell z-interference program

## Abstract

With gate length (Lg) and gate spacing length (Ls) shrinkage, the cell-to-cell z-interference phenomenon is increasingly severe in 3D NAND charge-trap memory. It has become one of the key reliability concerns for 3D NAND cell scaling. In this work, z-interference mechanisms were investigated in the programming operation with the aid of Technology Computer-Aided Design (TCAD) and silicon data verification. It was found that the inter-cell trapped charges are one of the factors causing z-interference after cell programming, and these trapped charges can be modulated during programming. Thus, a novel program scheme is proposed to suppress the z-interference by reducing the pass voltage (Vpass) of the adjacent cells during programming. As a result, the proposed scheme suppresses the Vth shift of 40.1% for erased cells with Lg/Ls = 31/20 nm. In addition, this work further analyzes the optimization and balance of program disturbance and z-interference with the scaling of cell Lg-Ls based on the proposed scheme.

## 1. Introduction

**Figure 1:**
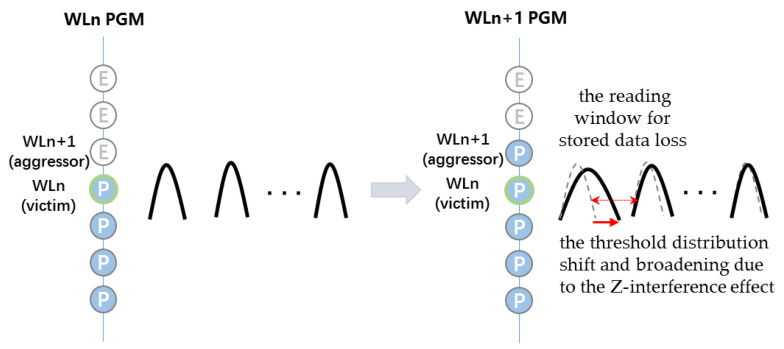
Schematic diagram of z-interference’s impact on the 3D NAND device’s reliability.

Due to the rapid development of the information era, 3D NAND memory is widely used in various applications to fulfill the explosive growth in data demand due to its good product performance and cost [1,2]. In the future development of 3D NAND, cell pitch shrinkage will be the inevitable and important way to increase storage density [3,4]. There is a prominent, non-ideal effect, called z-interface, in 3D NAND flash memory. The manifestation of this non-ideal effect is that the threshold voltage of the cell WLn (victim) is affected when the cell WLn + 1 (aggressor) is programmed, which manifests as the shift and broadening of the threshold distribution in the array operations, as shown in Figure 1. The reading window for stored data is affected by z-interference. With the cell Lg/Ls shrinkage, z-interference is one of the most critical concerns regarding device reliability [5,6,7]. In this situation, z-interference improvement is of great importance for the development of 3D NAND memory. In addition to process improvement [8], operation schemes have been reported to improve z-interference by adjusting the read voltage (Vread) of adjacent cells during the read operation [9,10,11]. These operation solutions used to improve z-interference mainly increase the WLn + 1 Vread voltage in order to increase the inverse electric field, thus reducing the WLn + 1 pattern’s impact on the channel barrier during WLn reading. However, there is a tradeoff between the read voltage tuning and read disturbance because of the higher Vread of the adjacent cell gate, which is also one of the key reliability requirements of 3D NAND flash [12]. Nevertheless, there are few studies on the improvement of 3D NAND z-interference during the cell programming operation.

In this work, the influence of the adjacent cell WLn ± 1 gate Vpass on z-interference during programming was investigated for the first time. With the aid of Technology Computer-Aided Design (TCAD) and silicon data verification, we propose reducing the adjacent cell WLn ± 1 Vpass to improve the z-interference during programming. A lower adjacent cell Vpass can reduce the inter-cell trapped electron density and suppress the channel barrier increase caused by the inter-cell trapped electron. The experimental data show that there is a 40.1% increase in the z-interference when the adjacent gate Vpass is reduced from 9 V to 3 V for a cell Lg/Ls = 31/20 nm in the erase pattern.

## 2. Methods and Principle

We used TCAD simulation to study the z-interference mechanism of 3D NAND memory and verify the principle with experimental test data. This is a common research mode in this field, as demonstrated by the reference articles [13,14,15]. The models used in TCAD device simulation are as follows: the Shockly–Read–Hall (SRH) model, non-local tunneling (NLT) model, Poole–Frenkel model and drift-diffusion model. These can effectively reflect the physical characteristics and have proven useful for explaining many phenomena of 3D NAND flash [16,17,18]. In addition, in order to simplify the research, this paper mainly studies the z-interference of WLn + 1 (aggressor) P7 pattern to the WLn (victim) erase pattern in the TLC mode, which is the worst case. The typical 3D NAND charge-trap memory device is a “junction-free” structure in which the transistors are connected without any junction between the adjacent cells. As an aspect of device dimension scaling, the cell threshold is more susceptible to, and influenced by, the program state of the adjacent cells [19].

**Figure 2:**
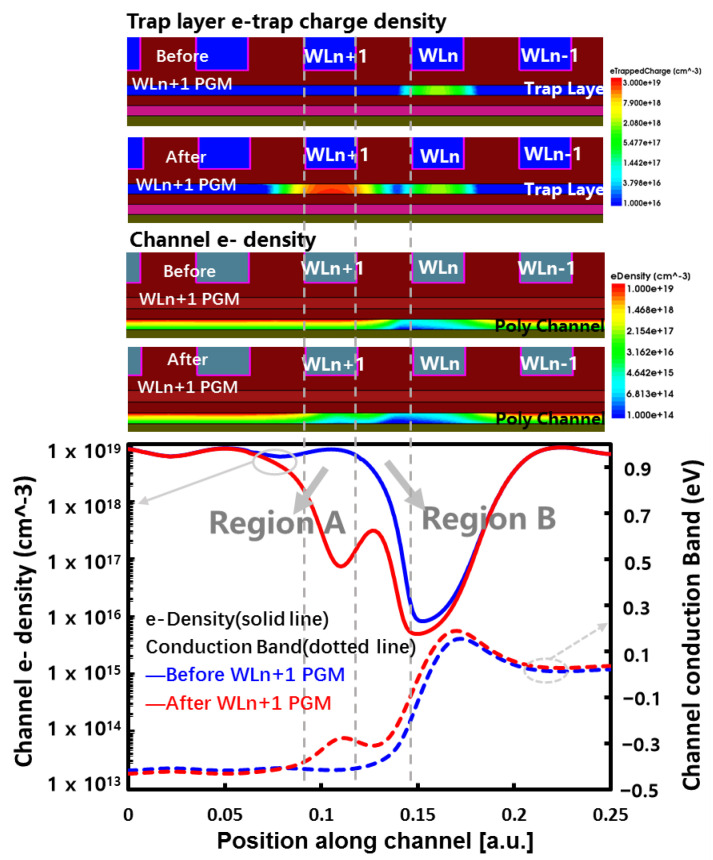
The z-interference simulation principal analysis.

Analyzing the z-interference mechanism of the simulation in Figure 2, it can be observed that when the selected WLn (victim) is read after WLn + 1 (aggressor) has been programmed, the channel inversion electron densities near the target cell are changed. For z-interference improvement, several works have aimed to decrease the channel barrier with operation schemes, but most studies have focused on the adjustment of the neighboring cells’ Vread during the read operation [9,10,11,16,20]. Other researchers have studied schemes of non-selected cells’ Vpass to improve the device characteristics during programming in 3D NAND flash, such as cell Vpass disturbance and PGM disturbance [21,22], but they have not clearly focused on the analysis of the influence of the adjacent cells’ WLn ± 1 Vpass on z-interference during programming. Additionally, there is no study on the components and formation factors of WLn (victim) channel barrier increase that arises during WLn + 1 (aggressor) pattern programming.

In this work, the channel was divided into different regions for z-interference analysis. Figure 2 mainly demonstrates the z-interference mechanism. As shown in Figure 2, the channel potential barrier, which is increased due to adjacent cell programming, can be divided into two regions: Region A and Region B. Region A corresponds to the WLn + 1 cell region. Region B is the inter-cell regime. For Region A, the increase in the channel potential barrier (the peak of the channel conduction band) is inevitable and irrelevant to the program pattern with certain device dimensions. Meanwhile, for Region B, compared with planar 2D NAND devices [23], the inter-cell charge is unique to typical 3D NAND trap charge memory devices, but it is not necessary for the formation of device patterns. It should be noted that z-interference can also be improved by suppressing the influence of Region B, which, to the best of our knowledge, has not yet been clearly studied. This work provides a further analysis of the effect of Region B on z-interference.

**Figure 3:**
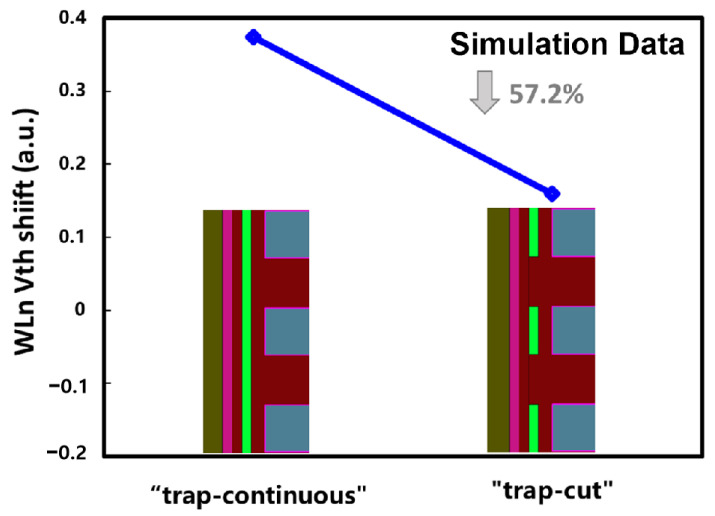
The z-interference comparison of the “trap-continuous” and” trap-cut” structures.

In order to confirm the influence of the Region B electron trap density on z-interference, TCAD simulation was carried out to compare the z-interference between the “trap-continuous” and “trap-cut” structures [24]. As there is no trapping layer between two adjacent cells in the “trap-cut” structure, the z-interference induced by the inter-cell charge is eliminated. The reading of WLn (victim) is affected only by the pattern of WLn + 1 (aggressor). The lower the Vth of WLn (victim), the higher the Vth of WLn + 1 (aggressor), and the greater the change in the channel barrier are, the more apparent the z-interference effect will be. In Figure 3, the simulation data show that the “trap-cut” structure has a 57.2% increase in z-interference as compared with the default “trap-continuous” structure. In other words, as expected, the z-interference can be improved by reducing the impact of the charge in Region B. Based on the above analysis, the trapped electronic charges in Region B are one of the reasons for z-interference in 3D NAND memory.

## 3. Proposal and Results

From the simulation analysis reported in the second section of this paper, we learn that the inter-cell charge in Region B is one of the key reasons for the z-interference, and the capture of these electrons is related to the electric field during the programming. The regulation of the adjacent cell Vpass is the most effective way to regulate this electric field; thus, we propose reducing the adjacent cell Vpass during the WLn + 1 (aggressor) programming phase to decrease the fringing field in the inter-cell region, thereby reducing the inter-cell charge in Region B to improve the z-interference.

**Figure 4:**
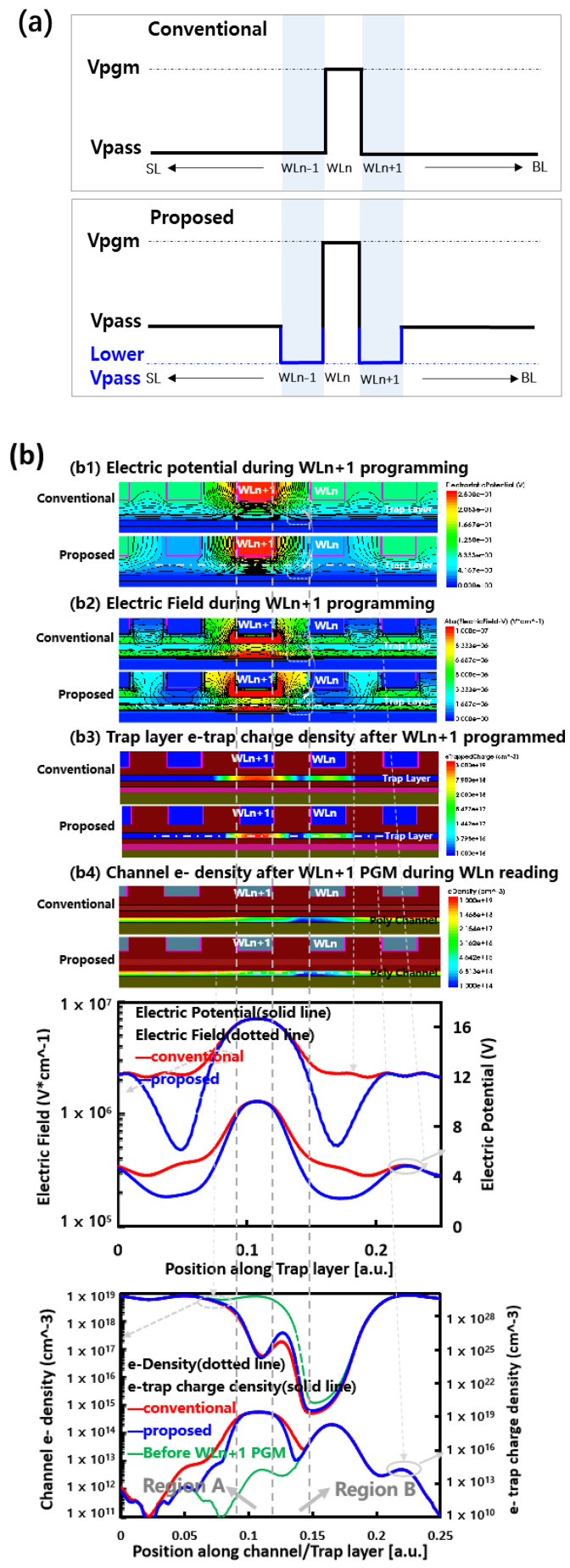

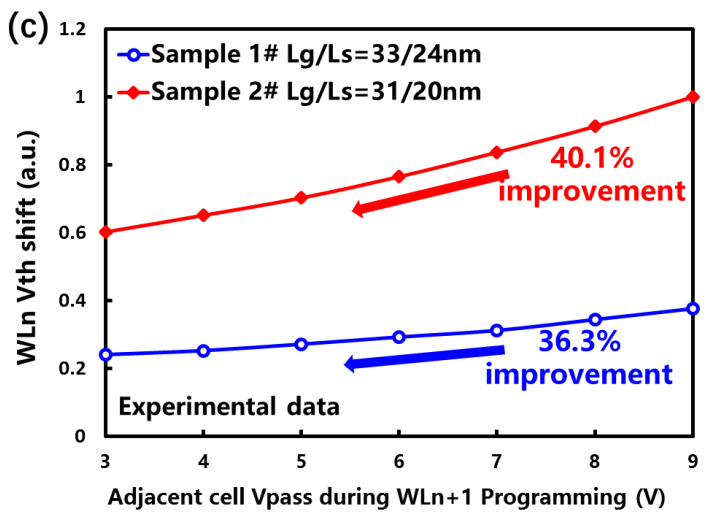
(**a**) Conventional and proposed scheme diagrams. (**b**) The simulation analysis of the proposed scheme. (**c**) The improvement of z-interference with the proposed scheme based on different Lg/Ls samples.

In contrast to Figure 2, Figure 4 demonstrates how to improve z-interference using the proposed scheme. Figure 4a shows the proposal scheme operation waveform diagram. Figure 4(b1,b2) shows the electric potential and electric field in the programming operation with the proposed scheme, demonstrating that the potential gradient between the programming cell and the adjacent cell is increased with the decrease in the adjacent cell Vpass, leading to an edge electric field distribution range decrease that enables FN tunneling to occur. Thus, after programming, as shown in the trap charge density analysis in Figure 4(b3), the proposed scheme reduces the inter-cell trap density, further reducing the Region B trap charge influence on the channel electrons’ inversion, shown in Figure 4(b4). Figure 4(b4) shows the change in the channel inversion electron concentration with the proposed scheme during WLn (victim) reading after WLn + 1 (aggressor) programming. Figure 4c shows the Vth shift caused by cell-to-cell z-interference during cell pitch scaling. The experimental data show a 40.1% improvement in the cell WLn + 1 (aggressor) P7 pattern to the WLn (victim) erase pattern with Lg/Ls = 31/20 nm due to the reduction in the Vpass of the adjacent cell from 9 V to 3 V during programming.

In summary, by simulating and analyzing the channel barrier and electron trap charge density in Region B, the inter-cell charge was confirmed to have an impact on z-interference. The proposed scheme reduces the adjacent cell Vpass in the programming operation for z-interference improvement. The underlying mechanism is the reduction in the inter-cell charge impact on the channel barrier increase by reducing the inter-cell electron charge density.

## 4. Discussion

**Figure 5:**
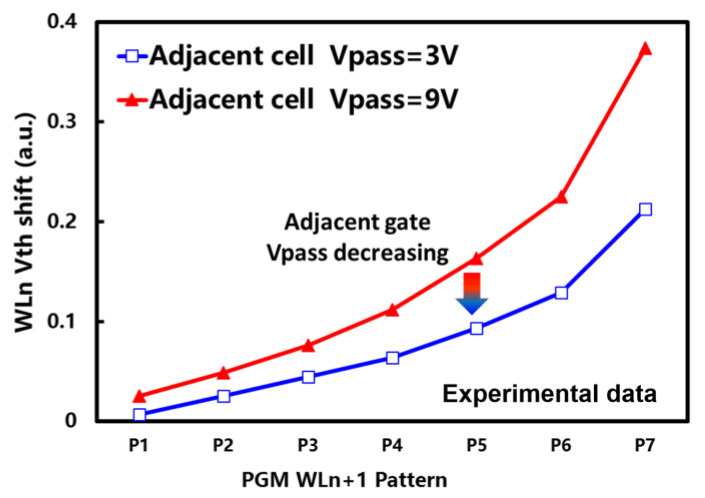
Comparison of different WLn + 1 (aggressor) patterns in regard to the decrease in the adjacent cell Vpass during programming to improve z-interference. P1 to P7 on the horizontal axis refer to different states in the TLC mode, which does not include the erase state.

This section provides a further discussion of the proposed scheme. The improvement of the proposed scheme was achieved by changing the electron trap charge distribution in the programming operation. For WLn + 1 (aggressor) programming according to different patterns in the TLC mode, there is always a suppression effect of the edge electric fields when reducing the adjacent cell Vpass. Generally, from the experimental data shown in Figure 5, we can see that the improvement of the proposed scheme will always be effective when the WLn + 1 (aggressor) is programmed according to different patterns.

It is worth mentioning that there is a drawback of adjacent cell Vpass reduction, which deteriorates the programming disturbance. The difference in channel boosting potential between the target cell and the adjacent cell will increase with the adjacent cell Vpass reduction in the inhibit string, leading to band-to-band tunneling and the hot electron injection effect [21].

**Figure 6:**
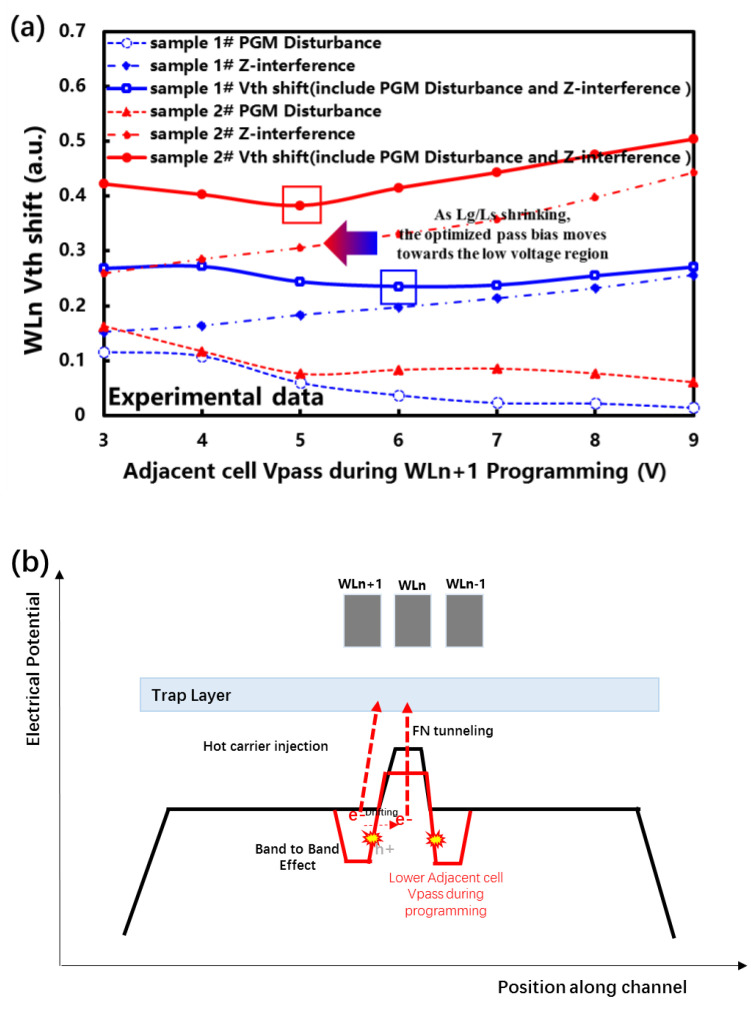
(**a**) Program disturbance and z-interference inducing Vth shift with different levels of adjacent cell Vpass (the blue lines indicate thicker Lg/Ls device electrical properties; the red lines indicate thinner Lg/Ls device electrical properties). (**b**) Explanatory diagram of the proposal scheme’s impact on the program disturbance.

As shown in Figure 6b, when the adjacent cell Vpass decreases, this will have an impact on the channel potential in the inhibit string. The electric field intensity between the target cell and adjacent cells will increase in the channel, which will enhance the band-to-band effect. Part of the hot electrons generated by the band-to-band effect will be injected into the trap layer due to the hot carrier injection effect, resulting in the deterioration of program disturbance. The other hot electrons will drift into the channel directly below the target cell under the action of an electric field, reducing the target cell’s boosting potential and increasing the potential difference between the channel and the gate. The free electrons in the channel below the target cell will be injected into the trap layer due to the FN tunneling effect, resulting in a further deterioration of program disturbance.

Therefore, there is an optimal condition for adjacent cell Vpass adjustment, considering the program disturbance and z-interference. In Figure 6a, the thick, solid line indicates the net increase in the total Vth shift, which comprehensively considers the z-interference and the program disturbance. With Lg/Ls shrinkage, the optimization point moves to the low-voltage region, which, due to the proportion of z-interference increase, influences the device characteristics’ degradation.

The differences between the two operation schemes in regard to z-interference improvement are listed and summarized below in Table 1. One of the schemes is based on the adjustment of the adjacent cell Vread during reading, which is the scheme reported in most articles [9,10,11], and the other scheme is based on the adjustment of the adjacent cell Vpass during programming, as proposed in this article.

Firstly, these two schemes are applied in different operations, with the former applied in the reading operation and the latter applied in the programming operation. Secondly, the principles of the two schemes are different. The former mainly enhances channel inversion by increasing the Vread bias in order to add an additional electric field, and the latter mainly reduces the negative effect of the inter-cell charge trap on channel inversion. Finally, the negative effects of the two schemes are also different. The former can cause a deterioration of the reading disturbance, but the latter can increase the programming disturbance. Based on these negative effects, there are also differences in the adjustable bias range.

It is worth mentioning that the improvement effects of these two schemes can be stacked without conflict. Compared to the conventional scheme, both of these schemes require independent control of the adjacent cell voltage during operation, which requires an additional voltage source. Thus, this scheme will increase the dynamic power consumption and entails a circuitry overhead (area). However, this increase is completely acceptable for NAND chip operation and circuitry design.

## 5. Conclusions

The z-interference is the most important factor affecting the device characteristics during 3D NAND cell shrinkage. The z-interference mechanism is the channel barrier increase observed after WLn + 1 (aggressor) programming. The inter-cell charge in the trap layer is partially responsible for the channel potential barrier increase, which can be modulated during programming. Thus, z-interference can be improved during the programming operation. This work clearly explains, for the first time, how the voltage of adjacent cells affects z-interference in 3D NAND devices during programming and proposes reducing the adjacent cell Vpass in the programming stage to decrease the inter-cell charge density. Considering the negative effect of Vpass reduction on program disturbance, there is an equilibrium point between the z-interference and program disturbance. As Lg/Ls shrinks, the optimized pass bias moves towards the low-voltage region due to the increased proportion of z-interference, impacting on the device characteristics.

## Figures and Tables

**Table 1:** Comparison of the schemes when adjusting the adjacent cell Vread and Vpass.

Z-Interference Improvement Schemes	Adjusting the Adjacent Cell Vread during Reading	Adjusting the Adjacent Cell Vpass during Programming
Operation	During Reading	During Programming
Principle	Adding an additional inverse electric field to enhance channel inversion	Reducing the negative effect of the inter-cell charge trap on the channel inversion
Voltage adjustment range	Smaller	Larger
Impact	Read disturbance	Program disturbance
Remark	The improvement effects of these two schemes can be stacked without conflict; both of these schemes require independent control of the adjacent cell voltage during operation, which requires an additional voltage source	

## Data Availability

Data are unavailable due to privacy.
